# Toward a Personalized Protocol Based on Skin Atrophic and Hypertrophic Photoaging Type With Injectable CaHA

**DOI:** 10.1111/jocd.70979

**Published:** 2026-06-08

**Authors:** Stefania Guida, Hassan Galadari, Nicola Zerbinati, Hugues Cartier, Steven P. Nisticò, Giovanni Pellacani, Ilaria Proietti

**Affiliations:** ^1^ School of Medicine Vita‐Salute San Raffaele University Milan Italy; ^2^ Dermatology Clinic IRCCS San Raffaele Scientific Institute Milan Italy; ^3^ College of Medicine and Health Sciences United Arab Emirates University Al Ain UAE; ^4^ Department of Medicine and Surgery University of Insubria Varese Italy; ^5^ Centre Médical Saint Jean Arras France; ^6^ Dermatology Unit, Department of Clinical Internal Anesthesiologic Cardiovascular Sciences Sapienza University of Rome Rome Italy; ^7^ Dermatology Unit Daniele Innocenzi, A. Fiorini Hospital Terracina Italy

**Keywords:** atrophic photoaging, biostimulation, calcium hydroxyapatite, hypertrophic photoaging, photoaging, wrinkles

## Abstract

**Background:**

Existing CaHA filler protocols for facial rejuvenation typically use skin thickness as a guide, but this parameter is difficult to measure and standardize in practice. Despite clear clinical differences between photoaging phenotypes, no previous studies have proposed a treatment approach based on these patterns.

**Objective:**

To evaluate the efficacy and safety of personalized CaHA protocols tailored to atrophic (AP) and hypertrophic (HP) photoaging types.

**Methods:**

A retrospective study assessed outcomes of CaHA treatments using pure (30%) or hyperdiluted (1:2) techniques, alone or combined, according to photoaging phenotype. Efficacy was evaluated at 6 months using validated mid‐ and lower‐face severity scales, Lemperle wrinkle scale, and Global Aesthetic Improvement Scale (GAIS). Safety was monitored throughout follow‐up.

**Results:**

Among 157 patients (mean age 62 ± 7.4 years), all severity scores improved significantly (*p* < 0.001), with overall severity decreasing from 16.4 ± 4.2 to 7.0 ± 2.2. HP patients achieved greater improvements in marionette lines, nasolabial folds, and Lemperle scores (*p* = 0.028), particularly with combined protocols, while AP patients benefited most in correcting sunken areas (*p* < 0.001) using pure CaHA. GAIS indicated high satisfaction (94% “improved” or better). No major adverse events occurred; minor effects resolved spontaneously.

**Conclusion:**

In this retrospective, practice‐based cohort, protocol selection aligned with photoaging phenotype, and the use of phenotype‐informed CaHA‐based approaches was associated with significant improvements in validated clinical scales and high patient satisfaction at 6 months. These findings reflect phenotype‐aligned practice outcomes, not comparative effectiveness. To confirm these results, prospective, randomized studies are needed, stratified by product type and balanced across phenotypes.

## 1 Introduction

Skin photoaging develops progressively, mainly from chronic ultraviolet (UV) exposure. While its earliest signs may appear in the third decade of life, it typically becomes clinically significant after the age of 40, when cumulative sun damage begins to manifest more distinctly [1]. Over time, photoaging diverges into two main phenotypes, atrophic and hypertrophic, each with unique structural, vascular, and clinical characteristics [2, 3, 4, 5].

Atrophic photoaging (AP) is characterized by sagging, fine wrinkling, erythema, and telangiectasia. These features reflect variations related to the extracellular matrix and increased vascular visibility, often associated with a higher risk of actinic damage and nonmelanoma skin cancers [2, 3, 4]. In contrast, hypertrophic photoaging (HP) presents with deep, coarse wrinkles and pronounced elastosis [2, 3].

The two phenotypes differ not only in appearance but also in their response to treatment. Advanced noninvasive imaging techniques—such as standardized evaluations with standard digital photography, optical coherence tomography (OCT), dynamic OCT (D‐OCT), and reflectance confocal microscopy (RCM) [6, 7, 8], have enabled a more precise characterization of these patterns, supporting the development of tailored therapeutic approaches [4].

Calcium hydroxylapatite (CaHA) is a biostimulatory filler with both volumizing and biostimulatory properties [9, 10, 11, 12, 13, 14]. Its versatility allows for use in both pure and diluted/hyperdiluted forms, depending on the clinical objective, when the goal is face contour or skin quality and tightness. Recent studies [5, 15, 16] have explored various CaHA protocols for facial rejuvenation, but there remains a need to align these approaches with the specific morphologic and functional features of photoaging subtypes.

We hypothesize, according to preliminary clinical observations, that tailoring CaHA protocols to photoaging type improves outcomes compared to a uniform approach. This exploratory study aims to evaluate the efficacy and safety of personalized CaHA filler protocols, using pure filler in the posterior approach or 1:2 hyperdilution or both modalities for the treatment of atrophic and hypertrophic photoaging.

## 2 Materials and Methods

This retrospective observational study included all consecutive patients ≥ 49 years (as per the definition of Sachs et al. [1] this is the age when photoaging type can be observed) who underwent facial rejuvenation treatments using calcium hydroxylapatite (CaHA)‐based fillers, treated between January and December 2024. Patients were classified into two groups—atrophic photoaging (AP) and hypertrophic photoaging (HP)—based on clinical evaluation and supported by imaging criteria described by Sachs et al. and Guida et al. [2, 3, 4]. Classification was guided by features such as erythema and dermal thinning for AP, and coarse wrinkling and dermal thickening for HP.

Exclusion criteria included allergy to local anesthetics, a history of connective tissue disease, prior skin rejuvenation treatments within 6 months before the study, previous mid‐ or lower‐face lifting procedures at any time, pregnancy or breastfeeding, ongoing immunosuppressive therapy, severe systemic diseases, and active skin lesions in the treatment area.

All patients provided written informed consent before treatment. The study was conducted per the Declaration of Helsinki, and all data were anonymized to protect patient confidentiality.

This manuscript adheres to the STROBE (Strengthening the Reporting of Observational Studies in Epidemiology) guidelines for cohort studies [17]. A completed STROBE cohort checklist is provided in Table S1.

### 2.1 Treatment Protocol

All patients were treated with CaHA‐based fillers using either pure or hyperdiluted protocols, depending on their photoaging phenotype. Two commercially available CaHA formulations were used: Radiesse (Merz, Frankfurt am Main, Germany) and HArmonyCa (AbbVie, North Chicago, Illinois, US), both of which contained 55.7% (weight/weight) CaHA as the primary biostimulatory agent, with particle size 25–45 μm [18]. Protocol selection (pure posterior vs. hyperdiluted/combined) was based on clinical judgment (e.g., phenotype‐related features, patient goals) and product availability, without randomization. Two CaHA‐based fillers were used: Radiesse (pure CaHA) and HArmonyCa (CaHA + cross‐linked HA). Protocol selection reflected product availability and clinical goals. Because the presence of HA in HArmonyCa can produce early volumization/hydration, product heterogeneity is treated as a key limitation. Outcomes were reported primarily at 6 months to reduce the influence of short‐term carrier/HA effects. Importantly, the study did not aim to compare the two products, but rather to evaluate the outcomes of CaHA‐based treatment approaches tailored to photoaging type.

This design reflected real‐world variability yet kept CaHA concentration and application consistent. Phenotype and technique were not independently randomized, limiting causal inference.

Pure CaHA‐based filler: the product was injected using a 22G, 5 cm cannula along the lateral aspect of the face, specifically posterior to the facial ligament lines [19, 20], to enhance lifting and provide structural support. The concept of neocollagenesis is based on previously published studies and was not directly assessed in this retrospective analysis [9, 10]. This technique aimed to restore dermal integrity and improve skin firmness in areas prone to volume loss. An overall amount of 1.5–3.75 mL of pure CaHA per patient was employed.

Hyperdiluted CaHA technique: it encompasses the use of CaHA 1:2 with saline and lidocaine.

Injections were performed using a 25G cannula through three entry points: one on the zygomatic arch, one on the mandibular border, and one at the end of the nasolabial fold. A total of 1.5–3 mL CaHA was used for 1:2 CaHA hyperdilution. This approach targeted the medium and lower face, aiming to improve skin texture and elasticity through biostimulation without adding volume.

### 2.2 Data Collection, Outcome Measures

Demographic and clinical data were collected, including age, sex, Fitzpatrick skin type, and the presence of associated dermatologic conditions (hyperpigmentation, acne scars, vitiligo, rosacea). Treatment areas were categorized as “posterior” for pure CaHA, “hyperdiluted” CaHA, and “both”.

### 2.3 Efficacy

Photographs were taken before (T0) and 6 months after treatment (T1). Images were taken with the patient in a sitting position and standard light to ensure consistency. Each patient served as their own control for comparison. Conventional standardized clinical photographs were available for all patients. Photoaging type and efficacy were assessed by two independent dermatologists; in cases of uncertainty, a third investigator was consulted to reach consensus. All patients underwent standardized follow‐up evaluation at 6 months after treatment.

Efficacy was evaluated through the comparison of the clinical images at T0 and T1 combined with validated aesthetic scales, including the mid‐face and lower‐face severity scales, and the Lemperle wrinkle scale [21, 22, 23]. The scales included: upper cheek fullness (scale from 0‐full upper cheek to 4‐very sunken upper cheek), nasolabial fold (NLF) at rest, marionette lines at rest, oral commissures at rest, and jawline at rest scales (scales from 0‐absent to 4‐very severe), Lemperle scale for cheek lines (from 0 = no wrinkles to 5 = very deep wrinkles) [21, 22, 23]. Each feature was scored, and an average was calculated for each scale, along with an overall severity score for every patient. This multidimensional grading approach enabled a comprehensive evaluation of both volumetric and biostimulatory effects acting as skin quality improvement agents following different protocols for CaHA treatment. Additionally, variables associated with overall severity scores were tested.

Furthermore, patients were asked whether they felt the results looked natural at T1. While this is a subjective judgment, a “natural” appearance has been defined as a condition that did not show obvious signs of cosmetic intervention, no visible product or irregularities on the skin surface [24, 25].

Aesthetic outcomes were assessed using the Global Aesthetic Improvement Scale (GAIS) at T1 and scored from 1 = very much improved, through 2 = much improved, 3 = improved, 4 = no change, up to 5 = worse. All patients were followed for 6 months after treatment.

### 2.4 Safety

Before starting treatment, the medical history of each patient was reviewed, and the area to be treated was examined. During the procedure, patients rated their pain on a scale from 1 to 10, with 1 being no pain and 10 being the worst imaginable. About 30 min after treatment, the area was checked for redness, swelling, or bruising. At every follow‐up visit, patients were asked about any side effects. These were categorized as either minor, requiring no medical treatment, or major, which included more serious issues like scarring, pigmentation changes, or granulomas that needed medical attention.

### 2.5 End Points

The primary efficacy endpoint of the study was to see whether there was at least a one‐point improvement on each of the severity scales, based on the dermatologists assessments of the before‐and‐after images. The safety endpoint was the absence of major side effects, requiring medical intervention.

### 2.6 Statistical Analysis and Biases

Statistical analysis was performed with SPSS statistical package 24.0 (IBM, Armonk, NY, US). Qualitative variables were expressed as absolute frequency and percentage, and compared with the chi‐squared test (Fisher's test for values ≤ 5). Quantitative variables were expressed as mean ± standard deviation (SD, range), and were compared with the T‐student's *t*‐test. The mean value was used as a cut‐off to categorize the variable into 2 classes, for exploratory categorization of quantitative variables in the regression analysis.

The association between overall improvement and different demographic factors, photoaging type, CaHA technique employed, product employed and associated skin conditions was tested. Significant variables from the univariate analysis were put into a multivariate regression model. A *p*‐value ≤ 0.05 was considered significant.

Potential biases included selection (non‐random protocol allocation), information (outcome measurement), and missing data. We mitigated selection and confounding by prespecifying covariates (age, sex, phototype, baseline severity, photoaging subtype, product, technique) and adjusting in multivariable models. Measurement bias was reduced by standardized photography and evaluations were to blinded treatment and identifiers. Missing data were summarized.

## 3 Results

### 3.1 Population

A total of 189 patients were treated with CaHA; of these, 157 were older than 49 years and, therefore, showed skin photoaging signs and were included in the current study, in accordance with Sachs et al. [1]. Baseline characteristics of the population were reported in (Table 1).

**TABLE 1:** Baseline characteristics of the population.

	Population *N* = 157
Age, mean ± SD (range)	62 ± 7.4 (49–76)
Sex, *n* (%)	
Females	133 (84.7)
Males	24 (15.3)
Phototype, *n* (%)	
1	22 (14.0)
2	89 (56.7)
3	35 (22.3)
4	9 (5.7)
5	2 (1.3)
Photoaging type, *n* (%)	
Atrophic	75 (47.8)
Hypertrophic	82 (52.2)
Treatment	
Pure posterior	72 (45.9)
Hyperdiluted	33 (21.0)
Both	52 (33.1)
Product	
Radiesse	41 (26.1)
Harmonyca	116 (73.9)
Skin comorbidities	
None	64 (40.8)
Hyperpigmentation	42 (26.8)
Rosacea	17 (10.8)
Acne scars	33 (21.0)
Vitiligo	1 (0.6)
Jawline T0	
1	4 (2.5)
2	56 (35.7)
3	46 (29.3)
4	51 (32.5)
Marionette T0	
1	8 (5.1)
2	45 (28.7)
3	52 (33.1)
4	52 (33.1)
Naso‐labial folds T0	
1	3 (1.9)
2	39 (24.8)
3	62 (39.5)
4	53 (33.8)
Sunken upper cheek T0	
1	100 (63.7)
2	32 (20.4)
3	14 (8.9)
4	11 (7.0)
Oral commissure T0	
1	5 (3.2)
2	50 (31.8)
3	51 (32.5)
4	51 (32.5)
Lemperle scale T0	
0	3 (1.9)
1	11 (7.0)
2	29 (18.5)
3	57 (36.3)
4	55 (35.0)
5	2 (1.3)

The mean age was 62 ± 7.4 years (range: 49–76), with predominantly female patients (84.7%). Fitzpatrick skin phototypes were mostly type II (56.7%), followed by type III (22.3%) and type I (14.0%). Patients were classified into atrophic (47.8%) and hypertrophic (52.2%) photoaging types. Treatment protocols included pure CaHA (45.9%), hyperdiluted CaHA (21.0%), and a combination of both (33.1%). Skin comorbidities were present in 59.2% of patients, with hyperpigmentation (26.8%) and acne scars (21.0%) being the most common (Table 1).

Baseline severity scores showed moderate to severe aging signs across facial regions, with the majority of patients scoring in the mid‐to‐high range on the jawline, marionette lines, NLFs, sunken cheeks, oral commissures, Lemperle cheek scale, and overall severity scale (Table 1).

As expected, older patients had higher baseline severity scores across most scales (*p* < 0.001), except for sunken cheeks (*p* = 0.425).

### 3.2 Efficacy

#### 3.2.1 Severity Scores

All scores showed significant improvement at T1 (after 6 months) compared to T0. Notably, the primary efficacy endpoint was met. The jawline score significantly decreased from 2.8 ± 0.9 at baseline (T0) to 1.1 ± 0.6 post‐treatment (T1) (*p* < 0.001). Similar reductions were noted for the marionette lines (2.8 ± 1.0 to 1.1 ± 0.6, *p* < 0.001), nasolabial folds (NLF) (2.9 ± 0.9 to 1.2 ± 0.5, *p* < 0.001), and sunken upper cheek (1.6 ± 0.9 to 1.0 ± 0.3, *p* < 0.001). The oral commissure score improved from 2.8 ± 0.9 to 1.2 ± 0.6 (*p* < 0.001), and the Lemperle score showed a reduction from 2.9 ± 1.1 to 1.2 ± 0.7 (*p* < 0.001). Overall, the total scale score significantly decreased from 15.7672 ± 4.48031 at T0 to 6.8624 ± 2.34115 at T1 (*p* < 0.001), confirming the efficacy of the intervention (Table 2).

**TABLE 2:** Distribution of severity mid and lower face scales, Lemperle scale, and overall severity scale (sum of all severity indexes), at T0 and T1, and *p*‐values according to T‐student's test.

	T0 (mean ± SD)	T1 (mean ± SD)	*p*
Jawline severity	2.9172 ± 0.88406	1.1847 ± 0.52893	< 0.001
Marionette lines severity	2.9427 ± 0.90753	1.1783 ± 0.56048	< 0.001
Naso‐labial fold severity	3.0510 ± 0.81489	1.2166 ± 0.49767	< 0.001
Sunken upper cheek	1.5924 ± 0.91956	1.0318 ± 0.26361	< 0.001
Oral commisure severity	2.9427 ± 0.87883	1.2229 ± 0.58396	< 0.001
Lemperle severity scales	2.9936 ± 1.02217	1.2102 ± 0.70740	< 0.001
Overall severity scales	16.4395 ± 4.20521	7.0446 ± 2.21111	< 0.001

All results at T1 were associated with a natural effect. Given the product heterogeneity and non‐randomized allocation, improvements are interpreted as within‐protocol and within‐product observations, rather than evidence of comparative superiority.

### 3.3 Variables Associated With Severity Scales Improvement

The analysis examined factors influencing clinical improvement. Univariate results showed significant associations for age (*p* = 0.007), skin involvement type (*p* = 0.001), treatment type (*p* = 0.009), and associated skin conditions (*p* = 0.007). No significant association between clinical improvement and product type was observed (*p* = 0.308).

These were further explored in a multivariate logistic regression showing the association of better overall scale improvement and skin photoaging type (OR = 72.6, confidence interval (CI) 95% 3.7–1434.9; *p* = 0.005), CaHA‐based approach (OR = 0.2, 95% CI 0.04–0.8; *p* = 0.025), and age (OR = 2.8, CI 1.2–6.1; *p* = 0.012). While the correlation between age and overall improvement in severity scales was expected, given that older patients presented significantly higher severity scores in aging scales at T0, the influence of other factors, such as skin photoaging type and the concentration of CaHA used, warranted deeper investigation.

The strongest finding was that patients with HP improved far more than those with AP. Additionally, the type of treatment influenced outcomes, and specifically, those receiving the “pure posterior” treatment had significantly better overall results than those receiving hyperdiluted or combined treatments.

However, this finding must be interpreted cautiously, as treatment was based on clinical judgment and patients expectations (i.e., contouring, skin quality), according to real‐life practice. Indeed, a total of 72 (96%) patients with AP were treated with pure posterior protocol, while 3 (4%) with AP were treated with hyperdiluted CaHA. On the other hand, 30 (37%) HP patients were treated with the hyperdiluted technique, and 52 (63%) of them were treated with both pure and hyperdiluted CaHA. The only feasible comparison was within the hyperdiluted subgroup (*n* = 33), where HP showed a higher rate of > 50% overall improvement than AP (96.7% vs. 33.3%; *p* = 0.017). This analysis is underpowered due to the small number of AP patients receiving hyperdilution (*n* = 3) and is reported for transparency rather than causal inference.

**FIGURE 1:**
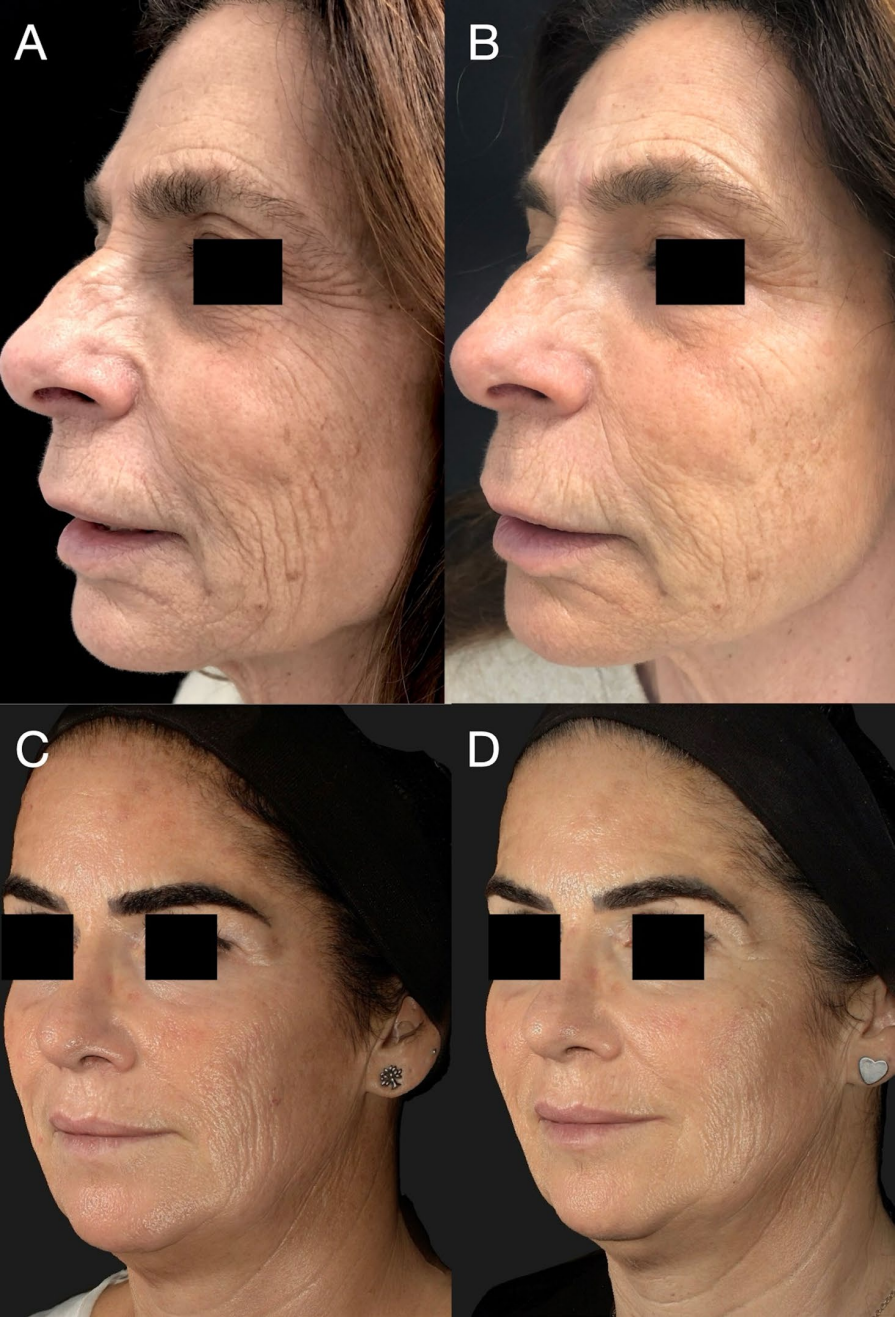
(A–D) Patients showing hypertrophic skin photoaging: (A) a 63 year‐old woman before treatment and (B) after 1.5 mL pure CaHA Radiesse plus (Merz, Frankfurt, Germany) treatment injected in the area posterior to the line of ligaments and 1.5 mL hyperdiluted 1:2 Radiesse classic (Merz, Frankfurt, Germany) of the mid and lower face. (C) A 53 year‐old woman before treatment and (D) 2.5 mL hyperdiluted 1:2 HArmoniCa (AbbVie, North Chicago, Illinois, US) of mid and lower face.

A subanalysis was conducted to explore which facial areas improved most according to photoaging type. HP patients showed significantly greater improvements in several key facial areas, including NLFs (*p* < 0.001), marionette lines (*p* = 0.001), oral commissures (*p* = 0.019), and the Lemperle cheek lines (*p* = 0.028) (Figure 1). These findings suggest that the more tailored approach used for HP patients, often involving a combination of pure and hyperdiluted CaHA, was particularly effective in enhancing contour and skin quality in these patients (Table 3).

**TABLE 3:** Severity scales improvement, overcoming 50% according to skin photoaging type.

	AP *N* = 75	HP *N* = 82	Total *N* = 157	*p*
> 50% improvement of jawline	30 (40.00)	45 (54.90)	75 (47.80)	0.062
> 50% improvement of marionette lines	29 (38.70)	53 (64.60)	82 (52.20)	0.001
> 50% improvement of NLF	30 (40.00)	57 (69.50)	87 (55.40)	< 0.001
> 50% improvement of sunken	18 (24.00)	3 (3.70)	21 (13.40)	< 0.001
> 50% improvement of the oral commissure	28 (37.30)	46 (56.10)	74 (47.10)	0.019
> 50% improvement of Lemperle scale	28 (37.30)	45 (54.90)	73 (46.50)	0.028

**FIGURE 2:**
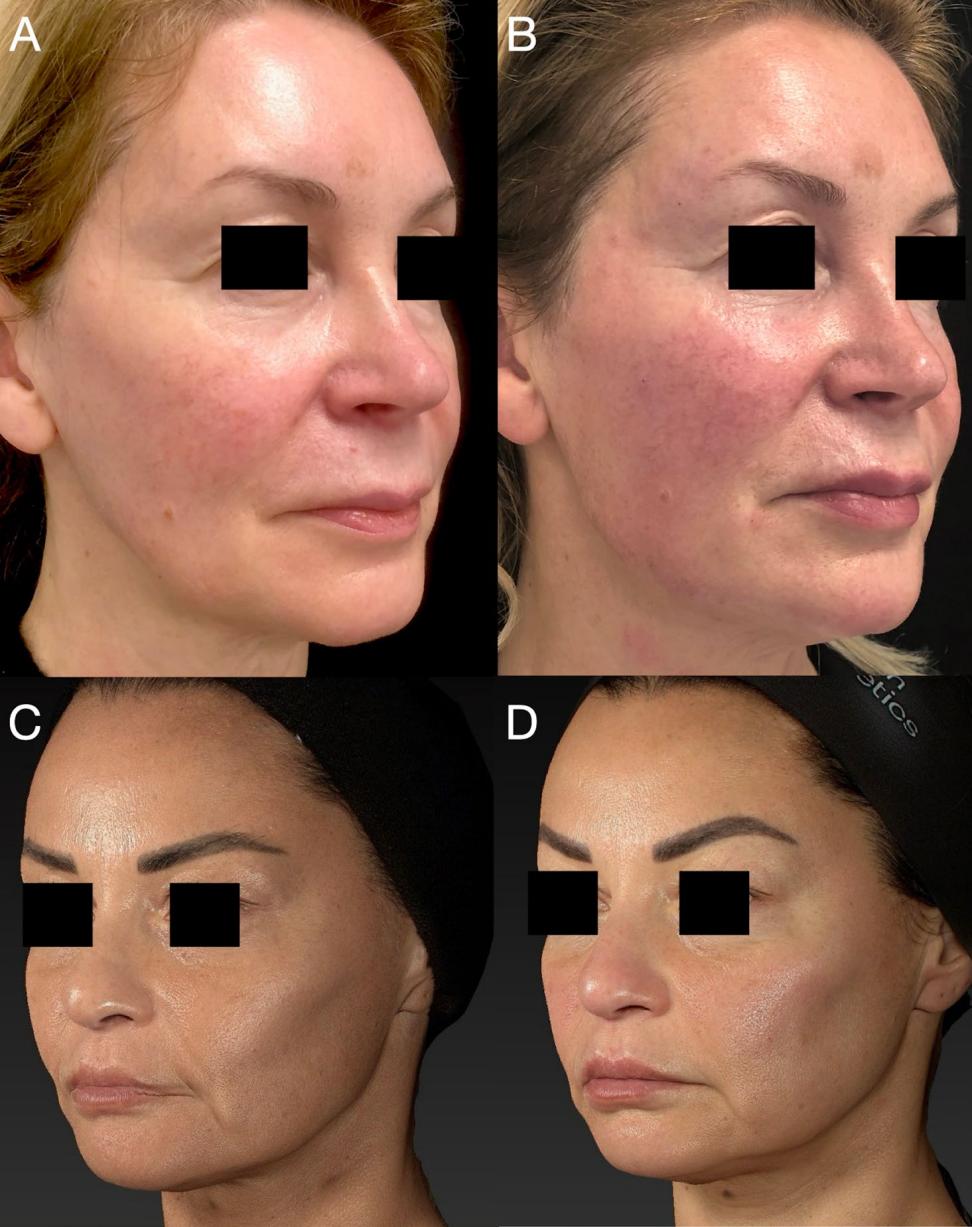
(A–D) Patients showing atrophic skin photoaging: (A) a 57‐year‐old woman before treatment and (B) after 1.5 mL pure CaHA Radiesse plus (Merz, Frankfurt, Germany) treatment injected in the area posterior to the line of ligaments. (C) A 50 year‐old woman, before treatment and (D) after 2.5 mL pure HArmoniCa (AbbVie, North Chicago, Illinois, US) treatment was injected in the area posterior to the line of ligaments.

Conversely, AP patients demonstrated significantly better outcomes in the correction of sunken areas (*p* < 0.001), likely due to the lifting effect of the pure posterior protocol predominantly used in this group (Figure 2; Table 3).

Finally, CaHA‐based approach was significantly associated with improvements in specific aging scales. The hyperdiluted technique was significantly associated with marionette lines (*p* = 0.003) and NLFs (*p* < 0.001) improvement, while pure posterior CaHA was associated with significant improvement of sunken cheeks (*p* < 0.001).

### 3.4 Satisfaction

The GAIS results reflect a high level of patient satisfaction following treatment. A total of 62 patients (39.5%) rated their improvement as “Very much improved,” 74 (47.1%) as “Very improved,” and 19 (12.1%) as “Improved”. Only 2 patients (1.3%) reported “No change,” and none reported worsening of their condition.

These findings align well with the clinical improvements observed across different skin photoaging types. Notably, the two patients who reported no change were among the three with atrophic AP treated using the hyperdiluted technique. This suggests potential benefits of tailoring CaHA protocols to photoaging phenotype, warranting further validation.

### 3.5 Safety

No severe adverse events were reported, thereby confirming that the safety endpoint was met.

Three minor adverse events were reported: erythema, edema, and ecchymosis. Erythema and edema occurred in all patients following the procedure and persisted for 2 to 3 weeks. Ecchymosis was observed in 25% of patients. All adverse events resolved spontaneously, without the need for additional treatment.

The average pain score was 1.3 ± 0.6 (range: 1–3) on a 10‐point scale.

## 4 Discussion

This study confirms that both efficacy and safety endpoints were successfully met for facial rejuvenation using CaHA‐based fillers. Personalized treatment protocols, whether using pure CaHA fillers with a posterior approach, hyperdiluted 1:2 CaHA, or a combination of both, proved effective in enhancing facial aesthetics across a diverse patient population.

Traditionally, treatment was guided by skin thickness [11, 12], a factor difficult to measure objectively in practice. Our results support a more refined, evidence‐based approach, tailoring treatment to phenotype, age, and injection technique. These factors were significantly associated with clinical improvement in multivariate analysis, marking a shift toward phenotype‐driven personalization.

Photoaging type emerged as a particularly strong predictor of treatment response. Patients with HP, characterized by coarse wrinkling, experienced significantly greater improvements in several facial areas, including areas of the medial face (such as NLFs and marionette lines). These improvements likely stem from combining pure and hyperdiluted CaHA. While pure CaHA may contribute to structural support, hyperdiluted CaHA is hypothesized to act predominantly at the dermal level [9, 10]. This interpretation is consistent with our previous noninvasive imaging data showing that wrinkles in HP subjects are associated with underlying dermal heterogeneity, supporting a rationale for dermal‐targeted biostimulatory approaches in addition to, or instead of, lifting‐based strategies [4].

Conversely, patients with AP, typically presenting with ligament‐related sagging rather than pronounced wrinkling, showed higher satisfaction with the pure posterior protocol, in this non‐randomized cohort. This technique, based on lateral injections placed posterior to the revised facial ligament lines, primarily targets ligamentous support and soft‐tissue repositioning while avoiding medial volumization [18, 19]. In this cohort, the approach was associated with significant improvement in sunken areas (*p* < 0.001), suggesting that mechanical lifting and structural reinforcement may be particularly relevant in AP.

Taken together, these observations support the clinical utility of modulating the CaHA‐based approach according to the predominant photoaging phenotype. Pure CaHA, administered in a posterior and lateral plane, may preferentially address ligamentous laxity and global support [17, 18] whereas hyperdiluted CaHA may exert its effects primarily within the dermis, contributing to improvements in skin quality and wrinkle appearance. Although dermal remodeling and biostimulation are inferred from established mechanisms and prior studies [9, 10] rather than directly demonstrated in the present analysis, the differential responses observed across photoaging phenotypes are consistent with a substrate‐driven treatment rationale. This mechanistic distinction may help explain why a uniform injection strategy is unlikely to achieve optimal outcomes across different photoaging patterns.

Existing literature consistently highlights CaHA's versatility as a biostimulatory agent [9, 10, 11, 12, 13, 14, 15]. While its volumizing effects are well established, increasing attention has been given to its regenerative properties, particularly when used in diluted or hyperdiluted formulations [26]. Studies have shown that CaHA, when appropriately diluted, can significantly improve skin quality, elasticity, and texture across various facial zones. Hyperdiluted CaHA (commonly at a 1:2 ratio) has demonstrated notable improvements in validated aging scales, with high levels of patient satisfaction and a favorable safety profile [10, 15, 16]. These findings, coming from the literature, reinforce the role of CaHA not only as a volumizing agent but also as a powerful tool for dermal remodeling and skin rejuvenation [9, 10, 15, 16, 26].

Beyond the specific techniques evaluated, the phenotype‐guided CaHA protocols described here may be conceptually positioned within contemporary multimodal facial rejuvenation strategies. Modern aesthetic practice increasingly relies on the coordinated use of structural fillers, biostimulatory agents, neuromodulators, and energy‐based technologies to address the multiple anatomical layers involved in facial aging. Within this framework, a phenotype‐driven CaHA approach may contribute to treatment planning by aligning the mechanism of action of the product with the predominant aging substrate.

Although diluted and hyperdiluted CaHA are widely used, randomized controlled trials evaluating these techniques are still lacking [15]. This highlights the need for further high‐quality studies to validate and refine dilution protocols, injection techniques, and treatment intervals.

While these findings are promising, several limitations must be acknowledged. The retrospective design introduces potential selection bias and limits control over confounding variables. Although treatment protocols were aligned with current clinical standards, the lack of randomization and blinding may have influenced both treatment decisions and outcome assessments. Additionally, the absence of histological or advanced imaging validation limits objective confirmation of neocollagenesis or dermal remodeling. Therefore, these mechanisms are discussed as based on previous literature [9, 10] and should not be interpreted as demonstrated outcomes of this study. The sample size, though balanced between AP and HP groups, may be underpowered for detecting subtle subgroup differences. This limitation is reflected in the multivariate analysis, where some associations—particularly the relationship between skin photoaging phenotype and overall scale improvement—showed very large effect estimates accompanied by wide confidence intervals (OR = 72.6; 95% CI 3.7–1434.9; *p* = 0.005), suggesting potential model instability and imprecision likely related to the limited sample size. Therefore, these estimates should be interpreted cautiously and primarily as hypothesis‐generating signals rather than definitive effect sizes. Finally, the inclusion of two different CaHA‐based products, although applied consistently, introduces heterogeneity that may limit the generalizability of our findings. Specifically, pooling a pure CaHA/CMC filler (Radiesse) with a CaHA + HA hybrid (HArmonyCa) creates potential confounding, particularly in the early post‐treatment phase when HA‐mediated effects may predominate. While our 6 month assessment helps mitigate this issue, it does not fully eliminate the possibility of differential product effects. Therefore, the results should be interpreted as practice‐based associations within a mixed‐product cohort, reflecting outcomes observed in phenotype‐aligned clinical practice rather than evidence of comparative effectiveness.

These results can be applied to older (≥ 49 years) [1] showing AP or HP, predominantly female, phototypes I–III, using phenotype‐guided CaHA protocols. Generalization beyond these conditions is reasonable but should account for phenotype mix, injector technique, product heterogeneity, and patient‐level modifiers (i.e., age, sex, baseline severity).

In conclusion, this retrospective, practice‐based study suggests that phenotype‐informed protocol selection is associated with improved clinical scores and satisfaction. These findings reflect outcomes observed in phenotype‐aligned clinical practice rather than evidence of comparative effectiveness. Prospective randomized studies are required to determine causality and to directly compare protocols within a product and patient phenotypes.

## Author Contributions

S.G. and I.P. contributed to conception and design of the work. H.G., N.Z., H.C., S.P.N., G.P. contributed to the acquisition, analysis, or interpretation of data for the work. All authors drafted and reviewed the work critically for important intellectual content.

## Funding

The authors have nothing to report.

## Ethics Statement

The study was conducted in compliance with Good Clinical Practice and the Declaration of Helsinki.

## Consent

All participants provided written informed and photo consent (in Italian).

## Conflicts of Interest

The authors declare no conflicts of interest.

## Supporting information


**Table S1:** STROBE statement checklist.

## Data Availability

The data that support the findings of this study are available from the corresponding author upon reasonable request.
